# P-421. Outpatient antibiotic prescribing for acute respiratory infections: An observational study in two pediatric hospitals of Nepal

**DOI:** 10.1093/ofid/ofaf695.637

**Published:** 2026-01-11

**Authors:** Suraj Bhattarai, Jaya Dhungana, Ajit Rayamajhi

**Affiliations:** Global Health Research and Medical Interventions Institute (GlohMed), Lalitpur 44700, Bagmati, Nepal; Global Health Research and Medical Interventions Institute (GlohMed), Lalitpur 44700, Bagmati, Nepal; Global Health Research and Medical Interventions Institute (GlohMed), Lalitpur 44700, Bagmati, Nepal

## Abstract

**Background:**

Antimicrobial resistance (AMR) has been recognized as one of the leading causes of child mortality in low-and-middle income countries. Diagnostic challenges is impacting antibiotic decisions in children, further fueling the burden of AMR. This study aimed to assess antibiotic use practice for acute respiratory infections (ARI) in outpatient setting and compare results between public and private facilities.

**Figure:**
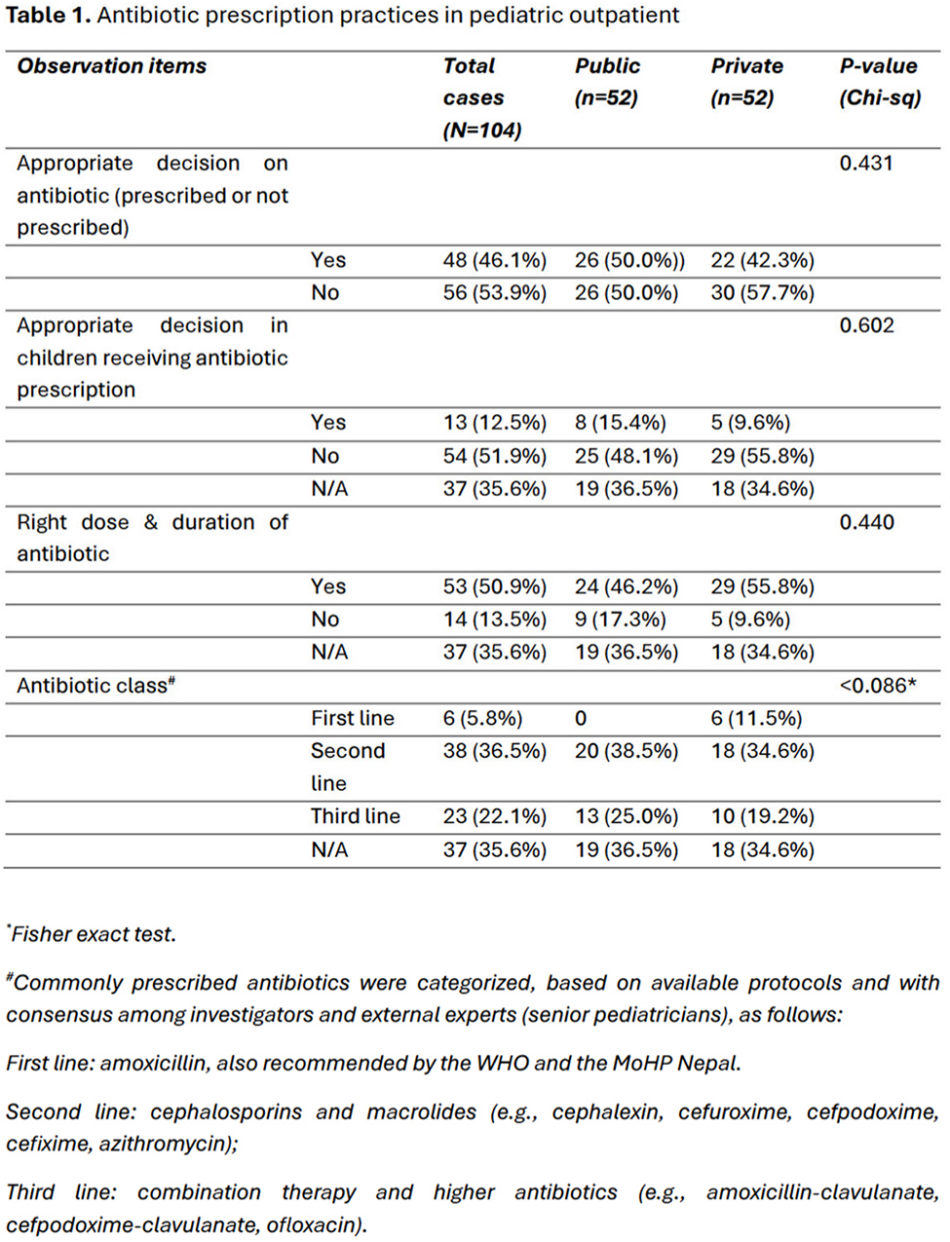

**Methods:**

A cross-sectional observational study was conducted in two pediatric hospitals of Nepal between January 2022 and July 2022. Data was collected using a standard observation checklist for outpatient management of under-5 children with ARI.

**Results:**

Out of 104 outpatient consultations assessed in public and private facilities (52 cases each), the majority (76%) of ARI diagnoses were based on the national protocol, and this practice was better in private facility than in public facility (85% vs 67%, p=0.039). Final diagnosis of ARI was reached after reviewing investigation reports in two-third of the cases; chest X-ray was ordered in 80%, complete blood counts in 57% and blood culture in 37%. Antibiotic was prescribed in 64% of the cases, with no difference between public and private facilities. Commonly prescribed antibiotics included azithromycin (28%), amoxicillin-clavulanate (25%), cefpodoxime (15%), amoxicillin (7%), cefixime (7%), and cefpodoxime-clavulanate (6%). Only 6% of the cases were given the first line antibiotics (amoxicillin), whereas, the majority received either second or third line antibiotics. The treating pediatrician’s decision on antibiotic (prescribed or not prescribed) was inappropriate in 54% of the cases, while among the cases that received antibiotics, the decision was inappropriate in 52%.

**Conclusion:**

This study found high proportion of inappropriate antibiotic prescription in pediatric outpatient for ARI, with higher tendency to use second or third line antibiotics. Facility-based pediatric antibiotic stewardship programs might be a useful approach to promote rational use of antibiotics for non-severe ambulatory cases in low-resource settings.

**Disclosures:**

All Authors: No reported disclosures

